# The Identification and Functional Analysis of mRNA Localizing to Centrosomes

**DOI:** 10.3389/fcell.2021.782802

**Published:** 2021-11-03

**Authors:** Hala Zein-Sabatto, Dorothy A. Lerit

**Affiliations:** Department of Cell Biology, Emory University School of Medicine, Atlanta, GA, United States

**Keywords:** RNA localization, centrosome, MTOC, co-translational tranport, post-transcriptional regulation, local translation, mRNA

## Abstract

Centrosomes are multifunctional organelles tasked with organizing the microtubule cytoskeleton required for genome stability, intracellular trafficking, and ciliogenesis. Contributing to the diversity of centrosome functions are cell cycle-dependent oscillations in protein localization and post-translational modifications. Less understood is the role of centrosome-localized messenger RNA (mRNA). Since its discovery, the concept of nucleic acids at the centrosome was controversial, and physiological roles for centrosomal mRNAs remained muddled and underexplored. Over the past decades, however, transcripts, RNA-binding proteins, and ribosomes were detected at the centrosome in various organisms and cell types, hinting at a conservation of function. Indeed, recent work defines centrosomes as sites of local protein synthesis, and defined mRNAs were recently implicated in regulating centrosome functions. In this review, we summarize the evidence for the presence of mRNA at the centrosome and the current work that aims to unravel the biological functions of mRNA localized to centrosomes.

## Introduction

To generate spatial enrichments of specific proteins, cells deploy a variety of strategies, including protein trafficking or local protein synthesis. RNA localization is the process by which mRNAs are enriched at subcellular locales. Often, RNA localization is coupled to translational control, whereby localizing RNAs are translationally repressed until they reach their final destinations. Thus, RNA localization offers an efficient means to generate spatially defined gene enrichments (Martin and Ephrussi, 2009).

Although perhaps best known for its role in developing embryos and oocytes, or highly polarized cells, such as neurons, RNA localization is a fairly ubiquitous post-transcriptional mechanism of gene regulation capable of altering acute cellular responses, like cell migration or division (Kislauskis et al., 1997; Groisman et al., 2000; Holt and Bullock, 2009; Katz et al., 2012). For example, the first observation of a localized mRNA was of *β-actin* mRNA in an ascidian embryo, later also found at the leading edge of migratory chicken fibroblasts (Jeffery et al., 1983; Lawrence and Singer, 1986). Many excellent reviews about RNA localization are available (Jansen, 2001; Martin and Ephrussi, 2009; Buxbaum et al., 2015; Das et al., 2021). Here, we wish to specifically address the topic of RNA localization to centrosomes. While the localization of RNA to centrosomes is now irrefutable, investigation into its biological significance is finally gaining momentum.

**FIGURE 1:**
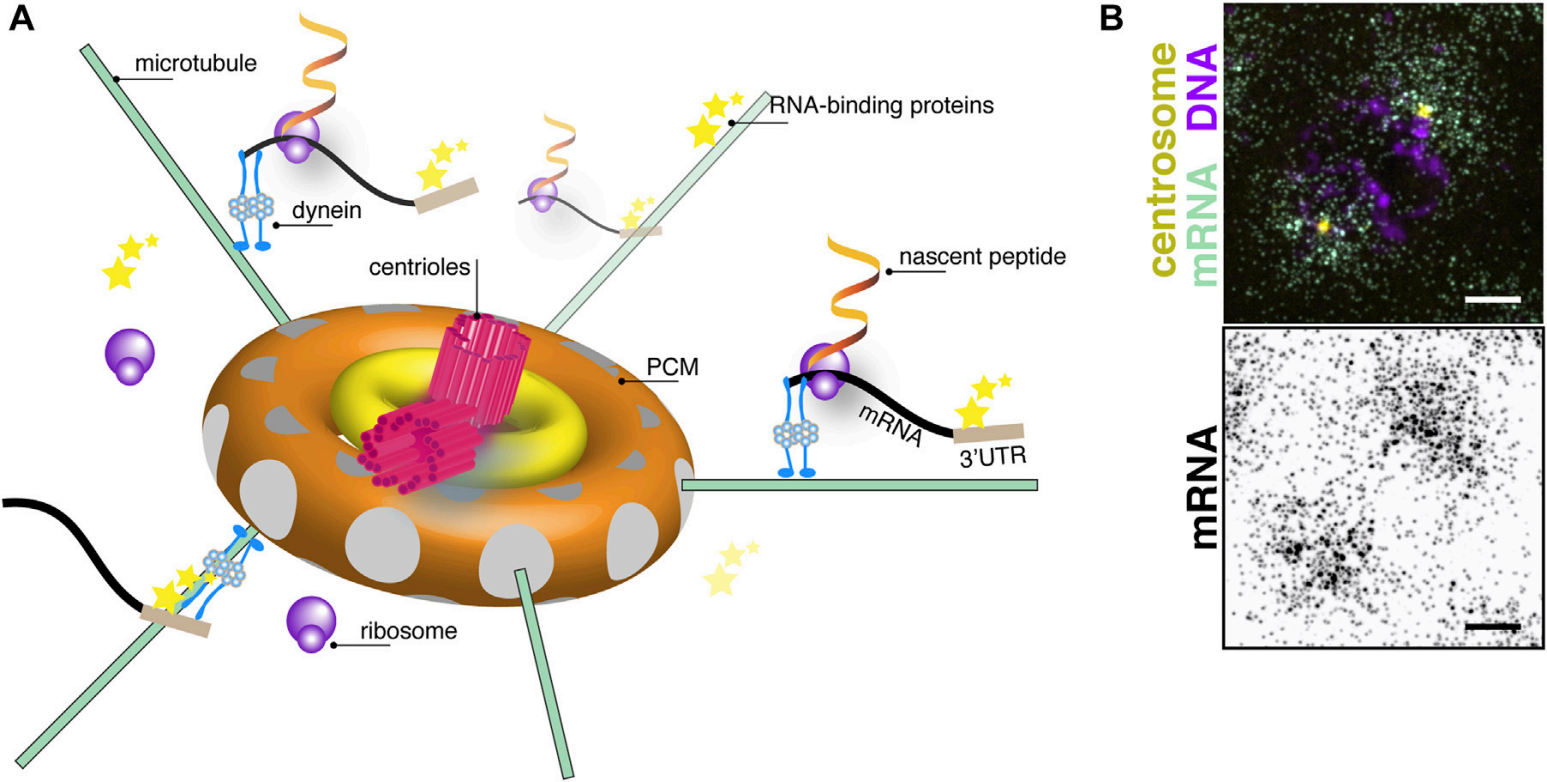
The centrosome as a center for translational control. **(A)** Cartoon schematic of centrosome organization showing a central pair of centrioles (magenta) with 9-fold radial symmetry of microtubule triplets surrounded by subconcentric rings of PCM (yellow and orange toroids). Microtubule filaments (green lines) are anchored with their minus-ends docked within *γ*-tubulin ring complexes (grey circles) embedded within the PCM. A model mRNA (black) recognized by RNA-binding proteins (stars) binding to its 3′-untranslated region (UTR; brown box) is shown undergoing active translation by ribosomes (purple circles). Nascent peptides (fiery ribbons) emerge near the centrosome. Note, objects are not drawn to scale. **(B)** Image shows single molecule fluorescence *in situ* hybridization for *plp* mRNA (green) in a prophase (DNA, magenta) syncytial *Drosophila* embryo. *plp* mRNA coalesces around centrosomes (yellow). Image below shows an inverted display of the mRNA channel to maximize contrast. Bar: 5 μm. Image courtesy of Dr. Junnan Fang, Ph.D.

The centrosome is a structured organelle comprising the centrioles, a pair of microtubule-based barrel-shaped structures at the center of the centrosome, and an encompassing protein matrix known as the pericentriolar material (PCM; Figure 1A) (Nigg and Raff, 2009). Although not restricted or compartmentalized by a membrane, the PCM is an organized yet dynamic structure that regulates centrosomal function as the primary microtubule-organizing center of most cells (Mennella et al., 2014; Woodruff et al., 2014). Importantly, cell cycle-dependent oscillations in centrosome structure and composition render the centrosome responsive to cellular demands (e.g., ciliogenesis) or developmental contexts (Khodjakov and Rieder, 1999; Palazzo et al., 2000; Nigg and Raff, 2009). Thus, centrosomes must modulate their activities rapidly, and RNA localization coupled to translational control represents one efficient means to do so.

The presence of nucleic acids at the centrosome held dubious importance despite several studies prior to the 2000s hinting at mRNA association with the centrosomes (for historical overview, we recommend (Marshall and Rosenbaum, 2000; Alliegro, 2011)). Progress in the field was hindered by conflicting results arguing about the incidence and relevance of mRNA at centrosomes. Early efforts to interrogate RNA at centrosomes typically involved 1) co-purifying RNA from isolated centrosomes, 2) detection of bulk RNA through non-specific labeling, or 3) treating cells or isolated centrosomes with RNase digestion and monitoring changes to centrosome size and/or microtubule nucleation. The “hammer” approach of RNase treatment led to contradictory findings (reviewed in Marshall and Rosenbaum, 2000), and progress in the field stalled. More refined and physiological approaches less prone to artifacts are now being deployed to systematically investigate defined mRNAs using a full suite of modern technologies. Recent work offers the titillating first glance at RNA function at centrosomes.

Why mRNAs reside at centrosomes is a question under active investigation. Increasing evidence, as we will discuss, supports the idea that some mRNAs are locally translated at the centrosome where their products are needed to tune centrosome activity. Still, other models are possible. For example, local RNA might contribute to centrosome structure or simply “hitch a ride” as a cargo to be passaged on to specific cellular lineages (Lambert and Nagy, 2002; Alliegro et al., 2006; Lerit and Gavis, 2011; Ryder and Lerit, 2018; Woodruff et al., 2018). While these remain intriguing possibilities, this review will focus on the centrosome as a center for translational control, as supported by recent advances.

## Delivering the Message: How RNAs Localize

RNA localization is an efficient means to generate local enrichments of protein activities, as a single mRNA may serve as a template for the synthesis of multiple translation products. Thus, localizing mRNA is more cost-effective in terms of cellular resources than localizing individual proteins. Further, because RNA localization is typically coupled to translational control, RNAs packaged within transport granules, or ribonucleoprotein (RNP) complexes, may be considered more inert as compared to their protein products, such as cell fate determinants, proteolytic enzymes, or other factors requiring precise, restricted activity (Das et al., 2021).

RNA localization occurs primarily through three distinct mechanisms: diffusion and entrapment, best exemplified by *nanos* mRNA in the *Drosophila* oocyte (Forrest and Gavis, 2003); protection from degradation, as typified by *Hsp83* mRNA in the *Drosophila* embryo (Ding et al., 1993); or active transport, as observed for many RNAs, including *ASH1* mRNA in budding yeast (Long et al., 1997; Takizawa et al., 1997; Bertrand et al., 1998). Active transport involves the trafficking of a cargo, such as an RNP, connected via adaptor proteins to molecular motors, which translocate on actin or microtubule cytoskeletal tracks (Bullock, 2011). Because centrosomes serve as sites of microtubule nucleation with microtubule minus-ends embedded within the PCM (Wu and Akhmanova, 2017), centrosomes are structurally suited as a hub for mRNA transport. RNA localization to the centrosome is, however, remarkably specific. As evidenced by genome-wide screens, relatively few transcripts reside at the centrosome (Lecuyer et al., 2007; Wilk et al., 2016; Chouaib et al., 2020; Kwon et al., 2021; Safieddine et al., 2021). The unique distributions of specific mRNAs rely upon *cis*-sequences, nascent peptides, and/or structural motifs within the RNA being recognized by *trans*-acting RNA-binding proteins. Multiple events through the RNA lifetime, including splicing, influence RNA localization patterns (Hachet and Ephrussi, 2004; Palacios et al., 2004).

## You’ve Got Mail: mRNA at the Centrosome

While non-specific labeling approaches and biochemical purification allowed early researchers to discover RNA at the centrosome, technological advances, including those in mRNA detection and transcriptomics, facilitated the identification of transcripts at the centrosome. Traditional *in situ* hybridization, which permits subcellular resolution of RNA distributions, remains a mainstay approach to localize mRNAs to centrosomes (Raff et al., 1990; Groisman et al., 2000; Lambert and Nagy, 2002; Kingsley et al., 2007; Lecuyer et al., 2007; Sepulveda et al., 2018; Bergalet et al., 2020). Single molecule fluorescent *in situ* hybridization approaches offer superior resolution and quantitative advantages (Figure 1B; Femino et al., 1998; Raj et al., 2008; Chouaib et al., 2020; Ryder et al., 2020; Ryder and Lerit, 2020; Kwon et al., 2021; Safieddine et al., 2021). Together, such approaches resolved the localization of specific mRNAs to centrosomes in diverse model systems, including *Ilyanassa*, *Spisula*, *Drosophila*, *Xenopus*, zebrafish, and mammalian cell lines (Raff et al., 1990; Han et al., 1997; Lambert and Nagy, 2002; Alliegro et al., 2006; Lecuyer et al., 2007; Sepulveda et al., 2018). Genetically encoded RNA aptamer tags, such as the MS2/MCP system, permit visualization of endogenous RNA dynamics in live cells (Bertrand et al., 1998). Recently, CRISPR/CAS-9 genome engineering was utilized to integrate MS2 stem loops into endogenous loci of the centrosome-localized *ASPM* and *NUMA1* mRNAs to monitor physiological and dynamic live trafficking. This study revealed *ASPM* and *NUMA1* mRNAs undergo directed transport toward centrosomes at velocities consistent with an active transport mechanism. Once localized, the RNAs remain anchored near centrosomes (Safieddine et al., 2021). As a whole, these localization-based approaches revealed several specific mRNAs are enriched at centrosomes in *Drosophila* and mammalian cells, showcasing the localization of mRNA to centrosomes as an evolutionarily conserved phenomenon.

While seeing is believing, transcriptomics approaches offer added insight for unbiased discovery of centrosome enriched mRNAs and subsequent bioinformatics analysis of shared features, including consensus motifs (Blower et al., 2007; Sharp et al., 2011). Presently, transcriptomics approaches remain relatively underutilized in identifying centrosome-specific mRNAs. Expanding transcriptomics approaches may uncover a consensus motif sufficient for mRNA targeting to centrosomes, or conserved motifs overrepresented in centrosome-localized RNAs. Nevertheless, recent advances in proximity RNA profiling, spatial transcriptomics, and related approaches will surely expand the parts list of mRNAs enriched at centrosomes (Jan et al., 2014; Li et al., 2018; Fazal et al., 2019; Alon et al., 2021; Engel et al., 2021; Rao et al., 2021). Once centrosome-associated mRNAs are identified, they must be validated by localization and, ideally, subjected to functional analysis.

## Prioritizing Messages in the Inbox

**FIGURE 2:**
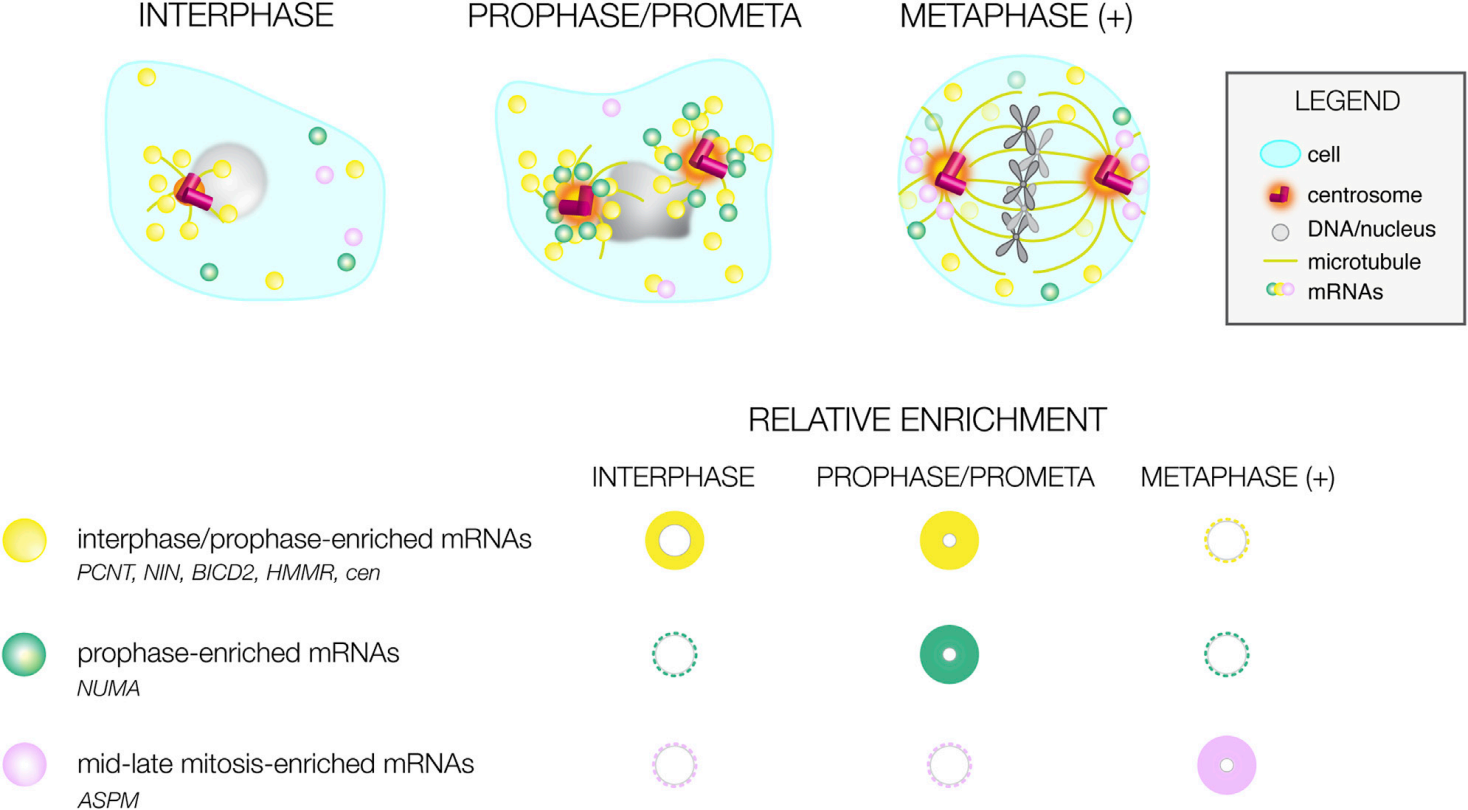
Cell cycle-dependent variances in mRNA distributions. Illustration of differential mRNA distributions of conserved centrosome-enriched mRNAs during interphase, prophase, and metaphase-to-late mitosis (metaphase (+)). Below, a graphical summary of mRNA distributions at the same cell cycle stages as reported by (Sepulveda et al., 2018; Ryder et al., 2020; Safieddine et al., 2021). The size and intensity of the circle correlates with the prevalence of mRNA localization; representative mRNAs are listed.

The use of genome-wide RNA localization screening strategies identified conserved mRNAs residing at centrosomes of divergent species (Table 1). Such transcripts should be prioritized for functional analysis, as the conservation of their localization argues for functional relevance. Many of these mRNAs show unique distributions that correlate with cell cycle stage, arguing RNA localization to the centrosome is a dynamic process (Figure 2). Most RNAs appear to preferentially enrich at prophase centrosomes, while relatively few RNAs reside at centrosomes during mid-to-late mitosis (metaphase (+), Figure 2). Moreover, these marked preferences for RNA localization to interphase/early mitotic centrosomes are conserved in human and *Drosophila* cells, perhaps to support the local synthesis of centrosomal proteins required before mitosis (Sepulveda et al., 2018; Ryder et al., 2020; Ryder and Lerit, 2020; Safieddine et al., 2021). Consistent with this idea, localization of RNAs to centrosomes generally precedes or correlates with the time at which they are translated (Tanenbaum et al., 2015; Safieddine et al., 2021).

**TABLE 1:** Conserved mRNAs localizing to centrosomes.

Gene name (synonyms)	Species	Sources
*abnormal spindle-like microcephaly associated* (*ASPM/asp*)	human, *Drosophila*	Sepulveda et al. (2018); Chouaib et al. (2020); Safieddine et al. (2021)
*BICD cargo adaptor 1* (BICD1/*BicD*)	human, *Drosophila*	Kwon et al. (2021); Safieddine et al. (2021)
*centrocortin (cen)*	Drosophila*	Lecuyer et al. (2007); Bergalet et al. (2020); Ryder et al. (2020)
*cyclin B* (*CCNB1/cyc B*)	*Drosophila, Xenopus*	Raff et al. (1990); Groisman et al. (2000); Blower et al. (2007); Lecuyer et al. (2007); Ryder et al. (2020)
*hyaluronan-mediated motility receptor* (*HMMR*)	human, *Xenopus*	Sharp et al. (2011); Chouaib et al. (2020); Safieddine et al. (2021)
*ninein* (*NIN*)	human, *Drosophila*, *Xenopus*	Lecuyer et al. (2007); Sharp et al. (2011); Kwon et al. (2021); Safieddine et al. (2021)
*nuclear mitotic apparatus protein 1* (*NUMA1*)	human, *Xenopus*	Blower et al. (2007); Chouaib et al. (2020); Safieddine et al. (2021)
*pericentrin* (*PCNT/PLP*)	human, *Drosophila*, zebrafish	Lecuyer et al. (2007); Sepulveda et al. (2018); Chouaib et al. (2020); Ryder et al. (2020); Safieddine et al. (2021)

Alphabetical list of mRNAs showing centrosome localization in two or more organisms from two or more independent studies, where “human” refers to human cell culture experiments. *, Centrosomal localization of *cen* mRNA is observed in multiple species of *Drosophila*: *melanogaster*, *simulans*, and *mojavensis* and to a lesser extent in *virilis* (Bergalet et al., 2020).

Additional conserved targets are informed by transcriptomic analysis of mRNAs associated with taxol-stabilized microtubules from *Xenopus* and cultured mammalian cells, which identified >100 common transcripts (Blower et al., 2007). Further, several of the transcripts identified as associating with taxol-stabilized microtubules are shared among those RNAs localizing to bona fide centrosomes, including *cyclin B1* (*CCNB1/cyc B)*, *ninein* (*NIN*), *hyaluronan-mediated motility receptor* (*RHMMR/HMMR*), and *nuclear mitotic apparatus protein 1* (*NUMA1*) mRNAs, indicative of partial overlap among these datasets (Blower et al., 2007; Sharp et al., 2011; Pascual et al., 2020). Such congruency is consistent with dynamic, microtubule-dependent localization.

Among these conserved transcripts, the first mRNA identified at centrosomes was *cyc B* mRNA. Raff and coworkers detected *cyc B* mRNA associated with spindle poles in a microtubule-dependent manner and further localized *cyc B* mRNA to anucleated centrosomes within syncytial *Drosophila* embryos (Raff et al., 1990). Cyc B protein also localizes to centrosomes, where it is required for mitotic progression (Minshull et al., 1989; Murray et al., 1989; Murray and Kirschner, 1989; Hagting et al., 1998; Huang and Raff, 1999; Wakefield et al., 2000). *cyc B* mRNA also resides at the spindle poles in early *Xenopus* embryos (Table 1).

The first clue that local mRNA influences centrosome function came from manipulating the RNA-binding protein responsible for targeting *cyc B* mRNA to spindle poles, CPEB (cytoplasmic polyadenylation element binding), which binds to cognate CPE cites within the *cyc B* 3′UTR. These experiments revealed that altering *cyc B* mRNA localization to the centrosome causes spindle morphogenesis defects and mitotic delay (Groisman et al., 2000). Subsequent work revealed the CPE motif is over-represented in datasets of mRNAs enriched on *Xenopus* and human taxol-stabilized microtubules, raising the possibility that CPEB proteins regulate other centrosome-enriched target mRNAs (Blower et al., 2007). Consistent with this idea, CPEB proteins localize to centrosomes and regulate the expression and localization of the master regulator of centrosome maturation, Plk1 (Groisman et al., 2000; Eliscovich et al., 2008; Pascual et al., 2020). Depletion of *CPEB1* in cultured mammalian cells reduces total Plk1 protein expression and recruitment to centrosomes (Pascual et al., 2020). CPEB proteins likely regulate other centrosome-associated genes, including those with mRNAs residing at centrosomes, as suggested by high-throughput datasets obtained using *Drosophila* and mammalian cell culture systems (Stepien et al., 2016; Pascual et al., 2020).

## At Your (Postal) Service: RNA-Binding Proteins and Ribosomes at the Centrosome

Besides CPEB proteins, other RNA-binding proteins, ribosomes, and translation initiation factors also localize to centrosomes (Figure 1A). Further, some RNA-binding proteins are implicated in PCM maintenance or other centrosome functions.

One multifunctional regulator of RNA metabolism is the RNA-binding protein Gle1, best described for its role in mRNA transport (Alcazar-Roman et al., 2006; Weirich et al., 2006), but also implicated in translation initiation and termination through DEAD-box proteins (Bolger et al., 2008). Gle1 localizes to the centrosome and the basal body of cilia, colocalizing with Pericentrin (PCNT) and also required for the recruitment of PCNT and NIN proteins to the centrosome. Further, *gle1* depletion impairs microtubule organization and ciliary function (Jao et al., 2017). Given its established role in mRNA transport and translation, Gle1 may promote *PCNT* and *NIN* mRNA recruitment to the centrosome, as recent studies note enrichment of these two transcripts at the centrosome through a translation-dependent mechanism (Sepulveda et al., 2018; Chouaib et al., 2020; Kwon et al., 2021; Safieddine et al., 2021).

Although not particularly enriched at centrosomes, Fragile-X Mental Retardation Protein (FMRP) is an RNA-binding protein contributing to centrosome functions and normal mitotic progression. FMRP is encoded by the *Fmr1* gene, which, when mutated, is associated with Fragile-X Syndrome (FXS), the most common heritable form of intellectual disability and autism spectrum disorder (Santoro et al., 2012). Loss of FMRP results in disordered microtubules and altered microtubule-dependent intracellular trafficking, which likely influences the pathophysiology of FXS (Yao et al., 2011). Among the putative RNA targets of FMRP, several overlap with CPEB (Costa et al., 2005; Udagawa et al., 2013). FMRP regulates mitotic progression in many tissues, as loss of *Fmr1* leads to elevated rates of neural stem cell proliferation, resulting in impaired neurogenesis in *Drosophila* and, remarkably, in induced pluripotent stem cells (iPSCs) derived from FXS patients (Callan et al., 2010; Raj et al., 2021).

Recent work implicates FMRP as important for the regulation of the centrosome-localized *cen* mRNA in *Drosophila* (Ryder et al., 2020). *cen* mRNA was first localized near spindle poles by a genome-wide RNA localization screen (Lecuyer et al., 2007). Importantly, centrosomal localization of *cen* mRNA is conserved among various *Drosophila* species, despite millions of years of evolutionary distance (Table 1; Bergalet et al., 2020). Cen is required for embryonic development, as loss of *cen* impairs centrosome separation, spindle morphogenesis, and actin cleavage furrow formation, leading to embryonic lethality (Kao and Megraw, 2009). Concurrent work showed *cen* mRNA assembles into large pericentrosomal RNPs that colocalize with Cen protein during later syncytial embryonic stages (Bergalet et al., 2020; Ryder et al., 2020). Furthermore, puromycylation-proximity ligation assay (puro-PLA) experiments suggest Cen is locally translated near centrosomes (Bergalet et al., 2020). Loss of *Fmr1* increases the localization of *cen* mRNA to *Drosophila* embryonic centrosomes and enhances translation of Cen protein, suggesting FMRP functions to attenuate *cen* mRNA localization and translation. Consistently, reduction of *cen* dosage is sufficient to partially rescue mitotic spindle defects observed in *Fmr1* mutants; moreover, *cen* mRNA and protein associate with FMRP, implicating *cen* mRNA as an important FMRP target. Finally, mistargeting *cen* mRNA to the anterior cortex is sufficient to block *cen* mRNA and protein recruitment to distal centrosomes and recruits excess FMRP (Ryder et al., 2020). Mislocalized *cen* mRNA disrupts microtubule organization and induces elevated rates of mitotic errors, showcasing local dosage of *cen* mRNA as a key contributor to centrosome functions (Bergalet et al., 2020; Ryder et al., 2020).

In *C. elegans*, the RNA-binding protein SZY-20 localizes to the centrosome, suppresses embryonic lethality of the PLK4 ortholog, *zyg-1*, and restricts centrosome size by impairing recruitment of the PCM components SPD-2, SPD-5, and *γ*-tubulin; ultimately limiting microtubule-nucleation (Song et al., 2008). Similar findings were observed for Ataxin-2 (ATX-2), a conserved RNA-binding protein associated with spinocerebellar ataxia in humans, which associates with SZY-20 (Stubenvoll et al., 2016). ATX-2 itself localizes to centrosomes and is required for mitotic spindle orientation and successful mitosis (Gnazzo et al., 2016). Further studies are needed to identify relevant mRNA targets of SZY-20 and ATX-2 to understand how they influence centrosome activity.

In response to growth factor stimulation, the RNA-binding protein Hu Antigen R (HuR) is phosphorylated and localizes to centrosomes, relaxing its repression of *cyclin A* mRNA translation and permitting centrosome amplification characteristic of cancer cells (Filippova et al., 2012; Filippova et al., 2015). The HuR model is one example of how RNA-binding proteins influence centrosomal function by recruiting and stabilizing mRNA at the centrosome until needed for local translation.

Another major protein complex influencing RNA localization and converging on centrosomes is the exon junction complex (EJC). The EJC comprises three protein subunits (Magoh, EIF4A3, and RBM8A (Y14)) and mediates splicing, nonsense-mediated decay, RNA localization, and translation (McMahon et al., 2016). During mouse neurogenesis, loss of *Magoh* leads to errant mitotic spindle orientation in neuronal progenitors and spindle morphogenesis defects associated with incomplete centrosome separation. Similar phenotypes are observed by depleting other EJC components in cultured mammalian cells. Consequently, *Magoh* loss results in reduced neural stem cells and precocious neurogenesis, leading to microcephaly (Silver et al., 2010). Further, haploinsufficiency of either *Magoh*, *EIF4A3,* or *Y14* results in p53-dependent microcephaly in murine models (Mao et al., 2016).

Underscoring this pathophysiology is the localization of EJC components to centrosomes. In cultured mammalian cells, Y14 is significantly enriched at centrosomes (Ishigaki et al., 2014). Likewise, in mouse neural stem cells, EIF4A3 and Y14 localize to the basal body at the base of primary cilia in a microtubule and dynein-dependent manner (Kwon et al., 2021). Although both *BICD2* and *NIN* mRNAs associate with EIF4A3 and Y14 proteins and localize to the ciliary base, only *NIN* mRNA localization to the basal body is EIF4A3 and Y14-dependent in RPE1 cells. Depletion of *EIF4A3* and *Y14* also decreases localization of PCNT and *γ*-tubulin proteins, resulting in impaired microtubule organization and reduced ciliation, consistent with the spindle defects previously observed by Silver and co-workers (Silver et al., 2010; Kwon et al., 2021).

Proteomic analysis from isolated *Drosophila* centrosomes identified additional translational initiation factors, like EIF4A, and other RNA-binding proteins associated with centrosomes, such as poly(A)-binding protein (Muller et al., 2010). Consistent with a function in centrosome regulation, EIF4A localizes to centrosomes and its depletion impairs recruitment of PCM factors PCNT/PLP, Spd-2, and *γ*-tubulin, but not centriolar components. These phenotypes are likely unrelated to the role EIF4A plays in translation initiation because disrupting initiation by deleting other members of the EIF4F complex or inhibiting translation elongation by cycloheximide did not diminish the PCM (Muller et al., 2010). Further studies are needed to uncover the mechanism by which EIF4A restricts centrosomal size. As EIF4A promotes expression of oncogenes in pediatric leukemia, understanding how it regulates centrosome activity may inform human disease mechanisms (Wolfe et al., 2014).

Components of the EIF4F initiation complex associate and colocalize with centrosomal OFD1. While OFD1 weakly binds mRNA, the presence of BICC1, an RNA-binding protein also found at the centrosome, allows OFD1 to mediate a stronger association between eIF4F via eIF4E and several centrosomal mRNAs that are implicated in ciliogenesis and renal cyst formation (Iaconis et al., 2017). These studies support the notion that centrosomes serve as hubs for translational control.

## Reading the Message: Translation at the Centrosome

The enrichment of ribosomes, mRNA, and translation machinery supports local translation at the centrosome and spindle poles, as evidenced by local puromycylated ribosomes and azidohomoalanine (AHA) to detect nascent peptides (Blower et al., 2007; Bergalet et al., 2020; Pascual et al., 2020). Why is the centrosome a translationally active site? To answer this question, we must first recognize that the centrosome is a center of cellular management. It plays a major role in organizing the microtubule network, nucleating the spindle fibers during cell division, and forming the basal body in ciliated cells. These functions depend on the size and composition of the PCM, which will go through stages of expansion and shedding depending on the cell cycle stage. Altering the composition of the PCM around the centrosome at the mRNA level may be how the centrosome smoothly transitions between its cellular responsibilities. Changing local mRNA levels and translational status is an effective and efficient method of control. When no longer required, the mRNA can then easily be shuttled away in a translationally repressed state or degraded until needed again.

In support of this hypothesis, ribosomes co-purify with microtubules (Goldman and Rebhun, 1969), and ribosomal proteins decorate centrosomes and spindle poles (Blower et al., 2007; Sepulveda et al., 2018; Chouaib et al., 2020; Pascual et al., 2020; Kwon et al., 2021). Persuasive evidence for polyribosomes located near centrosomes comes from ultrastructural analysis; for example, see figure 18 in (Sorokin, 1962) and Plate 10 in (Murray et al., 1965). Similar findings are noted in recent 3D focused ion beam scanning electron microscopy (FIB-SEM) renderings of centrioles and basal bodies (Xu et al., 2017; Muller et al., 2021). Some mRNAs, such as *cyc B* mRNA, localize to centrosomes independent of translation; indeed, even the localization of ribosomes to centrosomes is translation-independent (Blower et al., 2007). However, many centrosome-enriched transcripts rely upon the presence of intact ribosomes, as determined by puromycin-sensitivity, consistent with a co-translational transport mechanism. These include *PCNT, ASPM, NUMA1, HMMR, CEP350, NIN, BICD2,* and *CCDC88C* mRNAs in cultured mammalian cells (Sepulveda et al., 2018; Chouaib et al., 2020; Safieddine et al., 2021) and *cen*, *asp* (*ASPM*), *Girdin*, *mud* (*NuMA*), and *BicD* mRNAs in *Drosophila* (Bergalet et al., 2020; Safieddine et al., 2021). Co-imaging nascent peptides using the SunTag system along with endogenous, MS2-aptamer-tagged mRNAs beautifully demonstrates transport of active polysomes translating *ASPM* and *NUMA1* transcripts as they move towards the centrosome (Safieddine et al., 2021). The minus-end directed microtubule motor dynein is implicated in the transport mechanism for some of these mRNAs (e.g., PCNT; Sepulveda et al., 2018). How commonly mRNAs are co-translationally localized by dynein to the centrosomes is still an open question.

## Postscript

To date, the rate of discovering RNAs localizing to centrosomes far outpaces their functional characterization, which remains a key bottleneck in the field. Convincing evidence of a direct role for mRNA at centrosomes comes from mistargeting or misexpression analyses of *cyc B* and *cen* mRNAs (Groisman et al., 2000; Bergalet et al., 2020; Ryder et al., 2020). These experiments allow experimenters to decipher whether local mRNA or protein affect centrosome functions and should be expanded in future studies. Additional approaches, including the expression of non-translatable transcripts, deletion of identified zipcodes, and mislocalization of aptamer-tagged RNAs will likewise prove informative.

Advances in our ability to detect mRNA at the single-molecule level *in vivo*, manipulate mRNA localization, and characterize specific protein-mRNA complexes precipitated a recent explosion of research investigating mRNAs at centrosomes, leading to novel insights in a short time. It seems the biological function of mRNA at the centrosome is finally being recognized as a significant regulatory paradigm. However, much work remains to understand which RNAs reside at centrosomes, how they get there, and what, precisely, they are doing.
